# Longitudinal course of physical and psychological symptoms after a natural disaster

**DOI:** 10.3402/ejpt.v4i0.21892

**Published:** 2013-12-27

**Authors:** Lars Wahlström, Hans Michélsen, Abbe Schulman, Hans Backheden, Riitta Keskinen-Rosenqvist

**Affiliations:** Centre for Family Medicine, Karolinska Institutet, Stockholm, Sweden

**Keywords:** Physical symptoms, mental health, natural disaster, longitudinal studies, pseudoneurological symptoms

## Abstract

**Background:**

After disaster, physical symptoms are common although seldom recognized due to lack of knowledge of the course of symptoms and relation to more studied psychological symptoms.

**Objective:**

This study aimed to investigate the change in the reporting of different physical symptoms after a disaster, including possible factors for change, and whether psychological symptoms predict physical symptoms reporting at a later point in time.

**Method:**

A longitudinal study of citizens of Stockholm who survived the 2004 Indian Ocean tsunami. A total of 1,101 participants completed questionnaires on somatic symptoms, general distress, posttraumatic stress, exposure, and demographic details 14 months and 3 years after the disaster. Physical symptoms occurring daily or weekly during the last year were investigated in four symptom indices: neurological, cardiorespiratory, gastrointestinal, and musculoskeletal. We used generalized estimating equations (GEE) analysis to determine odds ratios for a change in symptoms, and pathway analysis to predict the influence of psychological symptoms on physical symptoms.

**Results:**

There was a general decrease of reporting in all physical symptom indices except the musculoskeletal symptom index. The change in the neurological symptom index showed the strongest association with exposure, and for women. General distress and posttraumatic stress at 14 months postdisaster predicted physical symptoms at 3 years.

**Conclusion:**

Physical symptoms were predicted by psychological symptoms at an earlier time point, but in a considerable proportion of respondents, physical symptoms existed independently from psychological symptoms. Physicians should be observant on the possible connection of particular pseudoneurological symptoms with prior adversities.

Physical health problems are common after a disaster (Yzermans, Van den Berg, & Dirkzwager, 2009). In some cases these problems result from direct physical injury, such as burns from fires, or fractures and cuts after earthquakes (Catchpole & Morgan, 2010). Even without injury, experiencing a disaster may be associated with subsequent medical conditions if the hormonal stress response is not downregulated when safety is restored (Dirkzwager, Van der Velden, Grievink, & Yzermans, 2007; Zaetta, Santonastaso & Favaro, 2012). In addition, it is a commonly held belief that disaster, like other types of trauma, results in increased perceptions of somatic distress. The exposure to adversity leads to a reaction where psychological experiences in the form of impressions, thoughts, and strong emotions intermingle with physical reactions, such as weakness, trembling, perceptions of pain, or gastrointestinal upset, triggered by the sympathetic component of the autonomous nervous system, and the hormonal stress-response through secretion of cortisol (Shalev 2003). Under these circumstances, some of these physical reactions may become more enduring, and at a later point in time some individuals may not even link such physical reactions to their experience of the disaster (Van den Berg, Grievink, Stellato, Yzermans, & Lebret, 2005; Yzermans, Van den Berg, & Dirkzwager, 2009). To the extent this is the case they may, if presented to a physician, be labeled as “medically unexplained symptoms.” Physical symptoms of non-organic causes are often unspecific and individuals frequently have symptoms from multiple organ systems, both in general (Creed & Barsky, 2004) and after a disaster (Van den Berg, Grievink, Stellato, et al., 2005). For the most part, these are regarded as signs of general distress (Fink & Rosendal, 2008). Some studies have found physical symptoms after disaster exposure to be dependent on symptoms of psychological distress (Norris, Slone, Baker, & Murphy, 2006; Van den Berg, Grievink, Van der Velden, Yzermans, Stellato, Lebret, et al., (2008).

Even outside of a disaster context, symptoms with no definite medical explanation are common. A Norwegian population survey showed that 23% of males and 32% of females had been bothered by ≥5 somatic symptoms in the last year (Haug, Mykletun, & Dahl, 2004), and primary care surveys report a prevalence of medically unexplained symptoms ranging from 16 to 36% of visits (De Waal, Arnold, Eekhof, & Van Hemert, 2004; Kroenke, 2003; Toft et al., 2005). An association between medically unexplained symptoms and psychological problems has consistently been reported, although for a considerable proportion of people, no such association can be confirmed (De Waal et al., 2004; Haug et al., 2004; Kirmayer, Groleau, Looper, & Dao, 2004). In studies focusing on medically unexplained symptoms, constellations of symptoms are often defined, but as far as we know, symptoms associated with specific types of trauma have only been discussed in studies concerning sexual abuse (Paras et al., 2009).

Almost without exception, the relatively few investigations of somatic symptoms in a longitudinal perspective show a decrease in the prevalence of symptoms in the years following a disaster (e.g., Dirkzwager et al., 2007; Escobar, Canino, Rubio-Stipec, & Bravo, 1992; North, Kawasaki, Spitznagel, & Hong, 2004). Nonetheless, these symptoms may persist for a long time and there are indications that they may have a longer duration than psychological problems after disaster (Nijrolder, Van der Velden, Grievink, & Yzermans, 2011). Furthermore, somatic symptoms that co-exist with posttraumatic stress disorder (PTSD) tend to last longer (Andreski, Chilcoat, & Breslau, 1998).

For this paper, we have used data on a group of survivors from Stockholm collected at two time points—1 and 3 years after the 2004 Indian Ocean tsunami. Upon returning to Sweden after the disaster, practically all survivors were registered by the police and could thus be reached at a later date. In an earlier study (Keskinen-Rosenqvist, Michélsen, Schulman, & Wahlström, 2011), we showed that psychological strain during the tsunami disaster was associated with more frequent reporting of physical complaints 14 months thereafter. Notably, 20% of respondents reported physical complaints exclusively. We expanded this study with a follow-up 3 years after the disaster.

The present study had two aims. The first was to investigate the change in the reporting of different physical symptoms, including factors associated with the change in symptoms, from 14 months to 3 years after the tsunami disaster. The second aim was to investigate whether psychological symptoms may predict physical symptoms at a later point in time.

## Method

### Participants

A questionnaire was mailed 14 months after the disaster (T1) to 4,283 eligible persons 16 years and older who lived in the county of Stockholm and who, according to police registration at the airport, had returned from SE Asia in the weeks following the tsunami.

A total of 1,939 people (45%) returned the questionnaire and of these, 1,505 people stated that they had been in an affected area at the time of the wave. At 3 years (T2), these 1,505 respondents received a follow-up questionnaire, which was returned by 1,101 individuals: 439 men and 662 women (response-rate 73%; men 68%; women 76%), who were included in the study. At both time points, non-response was significantly more common among younger age groups and among men. Additionally, at T2, non-response was more common among the intermediately educated and among those not having full-time jobs.

There were no systematic differences in response between T1 and T2 with regard to exposure or other background variables. The regional ethical committees approved the study.

### Measures

We collected background factors such as age, gender, educational achievement, living arrangements, full-time work before the tsunami, and whether participants had children with them on the journey. The questionnaire contained the following four items related to the types of exposure to the disaster: presence on the beach (including in the water) when the wave hit, experience of life threat, severe physical injury, or loss of a significant person (Wahlström, Michelsen, Schulman, & Backheden, 2008). Eight categories were created out of the possible combinations of items, based on the actual experiences of the survivors. Some categories were comprised of a single item, while others were comprised of a combination of items. For example, all those who were severely injured were both present on the beach and experienced life threat. The category of least exposure was constituted by all those who answered “none of the above” to the exposure items, but who nonetheless were present in the disaster-stricken area and to some extent exposed to devastation or other victims.

### Outcome measures

The Stockholm Somatic Symptom Checklist (SSSC), described in detail in Keskinen-Rosenqvist (Keskinen-Rosenqvist et al., 2011), comprises 21 items concerning somatic complaints. Responses to the question “Have you had any of the following complaints during the last 12 months?” were coded as “1” if symptoms were reported “several times per week” or “every day,” and as “0” for the remaining three response alternatives. According to a factor analysis, items were organized into four indices: neurological, cardiorespiratory, gastrointestinal, and musculoskeletal (Keskinen-Rosenqvist et al., 2011). Each index was dichotomized with the reporting of any symptom coded as “1,” and the absence of symptoms coded as “0.” The Cronbach's alphas for the different indices were .80, .82, .77, and .88, respectively, in data from T1.

The 12-item General Health Questionnaire (GHQ) was used to identify general distress over the past few weeks, with each item scored 0–3. The higher the score, the more distressed the respondent. Responses were dichotomized in accordance with the constructors’ original instruction (Goldberg & Williams, 1988), whereby ratings of 0 or 1 are coded as “0” and ratings of 2 or 3 as “1.” The cutoff threshold between 2 and 3 (range 0–12) corresponded to the 75th percentile in the sample. The Cronbach's alpha was .92 in data from T1.

The Impact of Event Scale Revised (IES-R) consists of 22 items and was used to assess posttraumatic stress symptoms (Weiss, 2004). The degree of distress in the last week in response to a specific stressor is rated for each item on a 5-point scale, ranging from 0 = *not at all* to 4 = *extremely*, giving a range of 0–88. For the IES-R, the cutoff was set at the 75th percentile, resulting in a threshold of 32/33, which corresponds well with the cutoff used in other published studies (Asukai et al., 2002; Creamer, Bell, & Failla, 2003). The Cronbach's alpha was .95 in data from T1.

### Data analysis

First, we performed descriptive statistics to see response patterns at both time points. Log linear analyses for repeated measures were applied to test interactions between somatic symptoms and psychological symptoms over time. Secondly, in order to analyze the change over time in the somatic symptom indices, we estimated and tested a model using generalized estimating equations (GEE), using the logit link function and assuming the covariance structure unstructured. GEE is often used in analyzing longitudinal data where traditional regression models are inappropriate, for example, were the units to be analyzed are not independent (Ghisletta & Spini, 2004). The explanatory capacity of each variable in the model was assessed in a type 3 analysis. The number of variables was reduced with backward elimination according to the *p*-value, in order to obtain a fair estimate of the odds for change in symptoms, with adjustment for other variables of interest.

First, we performed descriptive statistics to see response patterns at both time points. Log linear analyses for repeated measures were applied to test interactions between somatic symptoms and psychological symptoms over time. Secondly, in order to analyze the change over time in the somatic symptom indices, we estimated and tested a model using generalized estimating equations (GEE), using the logit link function and assuming the covariance structure unstructured. GEE is often used in analyzing longitudinal data where traditional regression models are inappropriate, for example, were the units to be analyzed are not independent (Ghisletta & Spini, 2004). The explanatory capacity of each variable in the model was assessed in a type 3 analysis. The number of variables was reduced with backward elimination according to the *p*-value, in order to obtain a fair estimate of the odds for change in symptoms, with adjustment for other variables of interest.

**Fig. 1:**
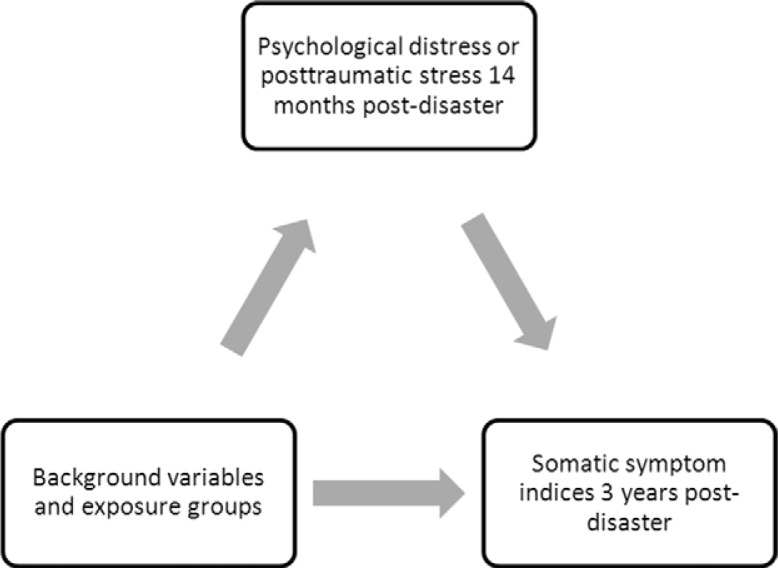
Hypothesized mediation model relating psychological distress and posttraumatic stress at 14 months, background variables and exposure groups with reporting in the somatic symptom indices at 3 years postdisaster.

Finally, we set up a pathway analysis in which the impact from GHQ or IES-R on different somatic symptoms was considered to be a mediator effect (Fig. 1). The explanatory variables used in modeling the mediator were gender, exposure groups and age groups. Since the mediator was dichotomous, we used Bayesian logistic regression, and pathway analysis based on the Markov chain Monte Carlo method (Huang, Sivaganesan, Succop, & Goodman, 2004; Li, Schneider, & Bennett, 2007). Before performing the pathway analysis, multiple regression analyses were performed to confirm that the direction and strength of associations between explanatory variables and physical symptoms were similar.

SAS 9.1.3 software (SAS Inst. Inc., Cary, NC) was used for statistical analyses, except for the pathway analyses where WinBUGS version 1.4.3. (WinBUGS 1996–2007, Imperial College School of Medicine at St Mary's, London) was used.

## Results

The proportion of respondents reporting physical symptoms decreased from 48 to 40%, and respondents reporting both physical and psychological symptoms decreased from 28 to 19% between measurements. 176 persons (16%) reported physical symptoms only at T1, whereas 94 persons (9%) reported physical symptoms only at T2. 71 individuals (6%) reported physical symptoms and posttraumatic stress symptoms at both time points. Table 1 shows the reporting of symptoms in the different indices for both T1 and T2. According to log linear analyses at both time points, women reported symptoms more often in all physical symptom indices as well as in the GHQ and the IES-R, compared to men.

**Table 1:** Number, proportions, and odds ratios (OR) for change of reporting in indices of Stockholm Somatic Symptom Checklist (SSSC), general distress (GHQ) and posttraumatic stress (IES-R), 14 months (T1) and 3 years (T2) postdisaster

		T1		T2		Change T1/T2^1^	
				
		n	%	n	%	OR	CI 95%
SSSC	Neurological	297	27	220	20	0.58^2^	0.45–0,74***
	Cardiorespiratory	110	10	73	7	0.56	0.38–0.83**
	Gastrointestinal	261	24	206	19	0.75	0.60–0.94*
	Musculoskeletal	332	30	287	27	0.89	0.72–1.09
GHQ		293	27	243	22	0.66^3^	0.51–0.85***
IES-R		262	24	132	12	0.37	0.27–0.51***

Note. SSSC, Stockholm Somatic Symptom Checklist, each index cut-off ≥1; GHQ: General Health Questionnaire 12, cut-off ≥2; IES-R: Impact of Events Scale Revised, cut-off ≥33.

1 All analyses adjusted for exposure group, gender and significant variables among age, educational level, living circumstances, pre-disaster employment, and company of children on the journey

2 Interaction Exposure group*Time *p*=0.07.

3 Interaction Exposure group*Time *p*<0.01.

* *p*<0.05,

** *p*<0.01

*** *p*<0.001.

In log linear analyses of response patterns over time, no physical indices interacted with GHQ in contrast to IES-R, where the interaction between time and each individual index was significant (with *p*<0.001 for every index). Table 1 also shows that in the GEE analyses with physical symptom indices as outcomes, there was a decrease in symptoms between time points in all indices except the musculoskeletal. With respect to co-factors for change, in the Neurological index only, there was an interaction with time for gender (*p*<0.01), and the number of symptomatic women decreased significantly (OR 0.46 CI: 0.35–0.59; *p*<0.001), in contrast to men (OR 0.73 CI: 0.53–1.02; *p*=0.07). In the Neurological index, the overall interaction Exposure group*Time approached significance (*p*=0.07), with a significant change in symptoms in two exposure groups [severe injury plus life threat plus presence on beach plus loss (*p*<0.01), and life threat only (*p*<0.05)].

In Table 2, the results of pathway analyses according to Fig. 1 shows that both GHQ and IES-R at T1 were predictors for outcome in all four somatic symptom indices at T2, when gender, age and exposure-groups were controlled for. Results from corresponding logistic regression analyses were similar, and consistently resulted in higher OR. Gender had a direct effect according to GEE analyses (data not shown), as well as an indirect effect via psychological symptoms according to pathway analyses.

**Table 2:** Odds ratios (OR) in a Bayesian path way analysis for general distress (GHQ) and posttraumatic stress (IES-R) at 14 months postdisaster as mediator variables predicting reporting in indices of Stockholm Somatic Symptom Checklist (SSSC), 3 years postdisaster

		Path analysis	
		
Outcome variable	Mediator variable	OR	CI 95%
Neurological	GHQ	3.8	2.7–5.3
	IES-R	3.8	2.6–5.4
Cardiorespiratory	GHQ	2.7	1.6–4.5
	IES-R	4.3	2.5–7.5
Gastrointestinal	GHQ	3.1	2.2–4.4
	IES-R	4.2	2.9–6.1
Musculoskeletal	GHQ	3.5	2.5–4.8
	IES-R	2.9	2.1–4.1

GHQ, General Health Questionnaire 12; IES-R, Impact of Events Scale Revised. Significant variables among background variables, including exposure groups, included in the statistical model.

Two separate questions concerned physical injury and were not part of the analysis. At T1, 64 (6%) individuals reported having sustained severe physical injuries in the tsunami. 22 and seven of these individuals reported at T1 and T2, respectively, that they still had major physical problems as a consequence. Due to the low numbers, we have assumed a negligible influence of physical injury on the reporting on the SSSC.

## Discussion

This longitudinal study of physical symptoms after a natural disaster found a general decrease in physical symptoms measured from 14 months to 3 years postdisaster. The decrease was retained after adjusting for co-factors for different symptom groups, with the exception of musculoskeletal symptoms. The change in the neurological symptom index showed the strongest association with exposure, and for women. We also found that the existence of physical symptoms at 3 years was predicted by general distress and posttraumatic stress at 14 months.

Yzermans and colleagues (Van den Berg, Grievink, Yzermans, & Lebret, 2005; Yzermans et al., 2009) have summarized studies of the course of physical symptoms after disasters, and point to the difficulty of making comparisons due to differences in disaster type, setting and methodology. They conclude that the prevalence of physical symptoms seems to decrease in the years after a disaster, but that the actual symptoms may nonetheless persist for a long time. Interestingly, we found no significant differences in change between physical symptoms and general distress, in contrast to posttraumatic stress where the reduction of symptom reporting was larger. This indicates that physical symptoms persist longer than psychological symptoms that are directly related to the disaster. Such a conclusion would be in line with a study of the Enschede fire disaster with the same time frame (Nijrolder et al., 2011).

With the exception of fatigue, there are few longitudinal studies that specifically examine the types of physical symptoms in disaster contexts. Spinhoven and Verschuur (2006) found very high prevalence rates of persistent fatigue in residents and rescue workers several years after a plane crash, and also an association with higher levels of psychopathology. In one of few studies with pre-disaster data, pseudo-neurological and gastrointestinal symptoms were more common 1 year after a flood and mudslide disaster in Puerto Rico (Escobar et al., 1992).

In this study, we found intriguing differences between the reporting of different kinds of symptoms. The items in the Neurological index stem from our clinical observation that individuals exposed to trauma often complain of mental fatigue, dizziness and clumsiness in addition to headaches. The importance of these types of symptoms was indicated by the strong significance for change between measurements. Furthermore, the interaction with exposure groups approached significance only with regard to neurological symptoms. Altogether, this implies that pseudo-neurological symptoms in particular should prompt the general practitioner or physiotherapist to interrogate further for the occurrence of potentially traumatic life events. Psychological treatment could then be offered as a complement to other forms of care.

It is a common notion that physical complaints resulting from stress or trauma are often manifested as musculoskeletal symptoms such as tense and aching muscles or physical weakness. Musculoskeletal symptoms have been reported as the most frequent and enduring type of symptom after disaster (Dirkzwager et al., 2007; Nijrolder et al., 2011). However, in the present study, the association between the disaster and these types of complaints was not significant. There may be several reasons for this. Musculoskeletal symptoms were the most frequent and at 14 months, the reporting of these may have already approached the symptom level of the general population. What is more, since musculoskeletal complaints are very common in the general population, changes in the frequency of these in our study group were difficult to detect.

Physical symptoms after a disaster may be a) a component of general distress or posttraumatic stress, or b) an independent phenomenon. Some longitudinal studies have found psychological symptoms (Bromet, Havenaar, Gluzman, & Tintle, 2005; Van den Berg et al., 2008), or PTSD-symptoms (Andreski et al., 1998; Dirkzwager et al., 2007; Norris et al., 2006), to mediate the effect of a disaster on somatic symptoms, although in one study the effect of PTSD-symptoms disappeared when other factors were controlled for (Spinhoven & Verschuur, 2006). More generally, in genetic and epidemiological studies, somatic symptoms have been shown to be partly independent from psychological symptoms (Creed et al., 2012; Kato, Sullivan, Evengard, & Pedersen, 2009). Our results showed that psychological symptoms may predict the emergence of physical symptoms in all our chosen dimensions, with consideration to gender, age and exposure. Still, 132 individuals (12%) reported physical symptoms but no psychological symptoms at both time points, and some of these individuals may have suffered from effects of the disaster. After the Enschede factory explosion, the majority of physical complaints were not reported to the general practitioner (Van den Berg et al., 2007). One could argue that physical symptoms are indeed common and often transient signs of stress. However, since the physical symptoms in our study were reported to occur at least several days per week throughout the last year, they ought not to be negligible.

In our study group, we could not confirm the conclusion of Kimerling and colleagues (2002) that exposure to trauma may attenuate gender differences in the reporting of physical symptoms. In contrast, women showed a prolonged recovery regarding neurological complaints in comparison to men.

### Strengths and limitations

First of all, one of the study's deficiencies is the late start of the first investigation phase, which was determined by practical circumstances. It is possible that this could have contributed to the lack of change in musculoskeletal symptoms between measurements. Although longitudinal, the study did lack a baseline, which is why we chose to investigate differences in change. In order to investigate the impact of psychological symptoms at T1 on somatic symptoms at T2, we chose not to use logistic regression analysis since both psychological and somatic symptoms are associated with the explanatory variables, that is, gender and exposure.

Since we were not able to collect information on medical diagnoses, theoretically, some of the reported physical complaints may in fact have been expressions of disease, and to the extent that this was the case, the associations for physical symptoms which we have found should be of less strength. However, given the probable good health of the study group, this should be of negligible importance. The lack of pre-disaster data was certainly a shortcoming since the existence of medically unexplained symptoms is a powerful risk factor for the emergence of such symptoms after disaster Van den Berg, Grievink, Van der Velden et al. (2008). An additional drawback was the lack of a proper instrument for registering depressive symptoms, since depression is associated with a number of somatic symptoms, particularly in the neurological sphere (Vaccarino, Sills, Evans, & Kalali, 2008).

Regarding the representativeness of the study group, it should be pointed out that the initial police registration included persons who returned from other parts of Southeast Asia, who were thus less motivated to respond. In addition, there is no reason to assume that our study group differed from the one in a Norwegian tsunami study, in which nonparticipants were less exposed and had fewer posttraumatic symptoms (Hussain, Weisæth, & Heir, 2009). Due to the underrepresentation of men among responders, one must be careful regarding conclusions for male survivors, as well as for intermediately educated or for younger age groups. A limitation to the generalizability of the results is the fact that, in contrast to many natural disasters, the studied group of survivors did not suffer damage to house and property, and returned to an intact society which mobilized to help them. Furthermore, due to the dominance of ethnic Swedes in the study group, findings should not be generalized to non-Western populations, since the expression of distress in psychological or somatic idioms is dependent on culture (Kirmayer et al., 2004).

Despite these potential limitations, the principal strengths of the study are the longitudinal design and the opportunity to study the effects of different types of exposure on a group of survivors with low levels of both pre- and postdisaster risk factors. Notably, there was no selection bias in relation to exposure categorization. The study contributes to current knowledge about disaster effects on physical symptomatology in two ways: First, it supports previous findings that psychological distress is a mediator for somatic symptom reporting at a later time point, and second, it highlights the importance of pseudo-neurological symptoms as a result of disaster experience. Finally, we believe this study merits attention because it links the frequent phenomenon of somatic symptoms in a non-patient context with adversities, and thus has implications for managing patients with medically unexplained symptoms in primary care.

## Conclusions

Physical symptoms postdisaster decreased with time. Physical symptoms were predicted by psychological symptoms at an earlier time point, but in a considerable proportion of respondents, physical symptoms existed independently of psychological symptoms. Physicians should be aware of the possible connection between physical complaints and prior adversities. The course of different types of physical symptoms after disaster, notably pseudo-neurological symptoms, merits further study.
