# Corrigendum

**DOI:** 10.3402/ejpt.v5.23877

**Published:** 2014-02-20

Regarding the paper ‘Longitudinal course of physical and psychological symptoms after a natural disaster’ by *Lars Wahlström, Hans Michélsen, Abbe Schulman, Hans Backheden, Riitta Keskinen-Rosenqvist*

Published in *European Journal of Psychotraumatology* 2013. Citation: European Journal of Psychotraumatology 2013, **4**: 21892 - http://dx.doi.org/10.3402/ejpt.v4i0.21892

The first name of one of the authors is wrong, namely Backheden, whose correct name is Magnus Backheden.

The correct list of authors are displayed below as well as the full abstract.

*Lars Wahlström, Hans Michélsen, Abbe Schulman, Magnus Backheden, Riitta Keskinen-Rosenqvist*

## Longitudinal course of physical and psychological symptoms after a natural disaster

### Lars Wahlström, Hans Michélsen, Abbe Schulman, Magnus Backheden and Riitta Keskinen-Rosenqvist

***Background***: After disaster, physical symptoms are common although seldom recognized due to lack of knowledge of the course of symptoms and relation to more studied psychological symptoms.

***Objective***: This study aimed to investigate the change in the reporting of different physical symptoms after a disaster, including possible factors for change, and whether psychological symptoms predict physical symptoms reporting at a later point in time.

***Method***: A longitudinal study of citizens of Stockholm who survived the 2004 Indian Ocean tsunami. A total of 1,101 participants completed questionnaires on somatic symptoms, general distress, posttraumatic stress, exposure, and demographic details 14 months and 3 years after the disaster. Physical symptoms occurring daily or weekly during the last year were investigated in four symptom indices: neurological, cardiorespiratory, gastrointestinal, and musculoskeletal. We used generalized estimating equations (GEE) analysis to determine odds ratios for a change in symptoms, and pathway analysis to predict the influence of psychological symptoms on physical symptoms.

***Results***: There was a general decrease of reporting in all physical symptom indices except the musculoskeletal symptom index. The change in the neurological symptom index showed the strongest association with exposure, and for women. General distress and posttraumatic stress at 14 months postdisaster predicted physical symptoms at 3 years.

***Conclusion***: Physical symptoms were predicted by psychological symptoms at an earlier time point, but in a considerable proportion of respondents, physical symptoms existed independently from psychological symptoms. Physicians should be observant on the possible connection of particular pseudoneurological symptoms with prior adversities.

Keywords: *Physical symptoms; mental health; natural disaster; longitudinal studies; pseudoneurological symptoms*

